# Community-Based Tuberculosis Preventive Treatment Among Child and Adolescent Household Contacts in Ethiopia

**DOI:** 10.3390/tropicalmed10040102

**Published:** 2025-04-09

**Authors:** Eshetu Abelti, Zewdu Dememew, Asfawesen Gebreyohannes, Yohannes Alemayehu, Tilay Terfassa, Taye Janfa, Degu Jerene, Pedro Suarez, Daniel Datiko

**Affiliations:** 1 USAID (United States Agency for International Development) Eliminate TB Project, KNCV (The Royal Netherlands Tuberculosis Association) Tuberculosis Foundation, Addis Ababa P.O. Box 1110, Ethiopia; eshetu.abdisaabelti@kncvtbc.org (E.A.); asfaw.gy@kncvtbc.org (A.G.); 2 USAID Eliminate TB Project, Management Sciences for Health, Addis Ababa P.O. Box 1157, Ethiopia; yalemayehu@msh.org (Y.A.); dgemechu@msh.org (D.D.); 3 National TB, Leprosy and other Lung Diseases Program, Addis Ababa P.O. Box 1234, Ethiopia; tilayegudina@gmail.com (T.T.); taye.letta@moh.gov.et (T.J.); 4 Division of TB Elimination and Health Systems Innovation, KNCV Tuberculosis Foundation, 2518 The Hague, The Netherlands; degu.dare@kncvtbc.org; 5 Management Sciences for Health, Global Health Systems Innovation, Arlington, VA 22203, USA; psuarez@msh.org

**Keywords:** community contact screening, household contacts, tuberculosis, TB preventive treatment, Ethiopia

## Abstract

There are limited studies on the community-based outcomes of tuberculosis (TB) preventive treatment (TPT) among children and adolescent contacts <15 years in Ethiopia. Our objective was to assess TPT uptake and completion rates among eligible under-15-year-old TB household contacts through an enhanced community-based model of interventions. The study was conducted between July 2021 and June 2022 in twenty primary health care units in the Sidama and Southern Nations, Nationalities, and Peoples’ Region (SNNPR) regions. A total of 4367 (99.2%) household contacts of 1069 bacteriologically confirmed PTB index cases were symptomatically screened for TB by trained health extension workers (HEWs) at the community level. A total of 696 (15.9%) symptomatic contacts were identified, of which 694 (99.7%) were evaluated for TB, resulting in 60 (8.6%) TB cases. A total of 1567 (95.3%) asymptomatic children and adolescent contacts <15 years of age were initiated on TPT (88.8%) at health posts in the community. After the interventions, there was a significant increase in contact screening coverage (95.6% vs. 99.2%, Odds Ratio (OR), 5.54; 95% Confidence interval (CI), 2.93–10.13) and TPT uptake (81.7% vs. 95.4%; OR, 4.67; 95% CI, 2.54–8.23). The TPT completion rate was also 98.1% (of 1567). The TPT completion rate at health posts in the community was higher than the rate at health centers (99.4% vs. 88.0%; OR, 20.95; 95% CI, 8.97–52.71). TPT uptake and completion in children and adolescent contacts could be improved remarkably with the implementation of an enhanced community-based model of intervention in high-TB-burden districts.

## 1. Introduction

Tuberculosis (TB) is a curable and preventable infectious disease; however, it remains a major global public health problem and is one of the leading causes of death worldwide [1]. People with prolonged exposure to infectious forms of TB can contract a TB infection, which often remains asymptomatic and non-infectious in the majority of those exposed [2]. About one-third of the world’s population is estimated to be infected with mycobacterium TB. If left untreated, 5–10% of people infected with TB develop active TB in their lifetime. Several factors, including immune-suppressive conditions, fuel the risk of progression from infection to disease. Household or close contacts of people with bacteriologically confirmed pulmonary TB (BCPTB) remain at greater risk of developing active TB without treatment of the TB infection. They may develop active diseases at any time during their lives [1,3,4]. Early provision of TB preventive treatment (TPT) for child and adolescent contacts is essential to reduce the risk of disease progression following recent exposure to infectious PTB [5].

TPT is highly efficacious and could prevent the development of active TB among high-risk groups by 60–90%, including in household contacts [3,6]. Scaling up TPT to these high-risk populations remain a priority intervention to meet the targets of the End TB strategy [3]. Access to short-course TPT regimens is a great opportunity for improved adherence and completion for at-risk population groups [7,8,9].

Globally, the overall progress of TPT uptake among household contacts has been less than 60%. Although TB programs are expanding TPT for children older than 5 years, coverage is still low. In a recent community-based study, TPT coverage for children 5–14 years ranged from 7.4 to 31.9% [10,11]. This shows that a substantial proportion of household contacts missed the opportunity to protect themselves [4]. In view of this, intensification and expansion of efforts and investments are required to accelerate active TB screening at the household level and improve the provision of short-course TPT [1]. Studies also support the critical need for innovative, community-based, and people-centered models of care to close the implementation gap in high-burden settings. The availability of shorter and child-friendly TPT regimens for child contacts provides an additional important opportunity to improve TPT acceptability and adherence [8,9].

Systematic contact investigation has been associated with both increased TB case detection and improved TPT uptake among contacts of people with BCPTB [3,6,8,9]. The cost-effectiveness of community-based contact investigation is well established. A recent cost-effectiveness modelling study in 12 high-TB-burden countries showed that household contact investigation with provision of short-course TPT has a value-for-money profile that compares favorably with other interventions, such as early TB case detection and treatment [12,13].

Contact tracing and investigation could be performed at a health facility, at the community level, or in both programmatic settings [3,6,9,14,15]. Several studies report that there are relatively lower rates of TPT initiation in facility-based passive contact tracing [9,15]. Other studies show improved TPT uptake of child contacts with intensified active and facility-based interventions for contact tracing at primary health care units (PHCUs) and hospitals [2,16,17]. However, in the facility-based model, the number of contacts identified and reached is usually limited, considering estimated targets around the index case. With passive contact tracing, a significant proportion of contacts may miss the opportunity to receive treatment [4,18,19]. There have been operational challenges in implementing contact investigation in programmatic settings. This could partly be explained by the fact that TB-exposed children are usually asymptomatic, and it may be difficult to bring them to health facilities for screening and subsequent TPT initiation. Other systemic and economic factors also contribute to underperformance in contact investigation, including TPT initiation [8].

In Ethiopia, the national TB control program recommends symptomatic screening and providing TPT to eligible household contacts <15 years of BCPTB cases once active TB is ruled out. Short-course rifamycin-based regimens—3 months of daily rifamycin and isoniazid (3RH) and 3 months of weekly rifapentine and isoniazid (3HP)—are indicated as preferred TPT regimen options to treat TB infection among contacts exposed to people with BCPTB [14]. Contact tracing and screening tend to be predominantly delivered at the health facility level using a passive approach, and there has been limited progress in reaching and providing TPT to eligible contacts with the current practice. Furthermore, as TPT services for children and adolescents between 5 and 14 years started in 2019 in Ethiopia, there is limited experience with TPT in this population group in programmatic settings. That is, the health system could not guarantee access to TB diagnosis, treatment, and prevention to the communities in resource-limited settings such as Ethiopia without an adequate community-based outreach framework. These issues were partly related to limited engagement and linkage of contact screening at community and health facilities with combined efforts and engagement of health care providers and community health workers.

Our objective was therefore to assess improvements in TPT uptake and completion among eligible under-15-year-old children and adolescent household contacts through the implementation of an enhanced community-based model of interventions at peripheral PHCUs in Ethiopia.

## 2. Materials and Methods

### 2.1. Study Setting

The study was conducted in ten districts of the southern regional states of Ethiopia during July 2021–June 2022. The Southern Nations, Nationalities, and Peoples’ Region (SNNPR) has an estimated population of 9 million. The region has 27 hospitals, 322 PHCUs, and 1713 health posts. Sidama regional state is an administrative region in Ethiopia established in 2021 and has a population of about 4.5 million. Previously, it was an integral part of the larger SNNPR. There are 22 hospitals, 135 PHCUs (health centers with satellite health posts), and 550 health posts in the region. According to national program reports, the annual TB case detection rate in these geographic areas usually exceeds 100%, suggesting high TB transmission in the community. Community TB care has been implemented by trained health extension workers (HEWs) at health posts in the districts as part of health extension program packages.

Two PHCUs from ten high-TB-burden districts in Sidama and SNNPR were purposely selected based on their relative volume of BCPTB cases enrolled in directly observed treatment (DOTs). One PHCU is estimated to serve a total population of 25,000. Enrolling more than 30 BCPTB cases was used as the criterion for designating a PHCU as high volume. The intervention was implemented in two phases (phase 1 in 8 PHCUs and phase 2 in 12 PHCUs). The preintervention periods were April–June 2021 (phase 1) and October–December 2021 (phase 2), while the intervention periods were July–December 2021 (phase 1) and January–June 2022 (phase 2). Twenty high-volume PHCUs were selected and engaged in the intervention.

### 2.2. Description of Interventions

TB contacts were determined as those individuals who shared the same living space with bacteriologically confirmed pulmonary TB cases for at least one night, or who had frequent or extended daytime exposure in the 3-month period prior to diagnosis. Thus, during household visits, the HEWs tend to list and assess household members, relatives, and friends who shared airspace at night, and interview the index case to trace those who had frequent exposures in the neighborhood and workplace. The intervention package of enhanced community-based contact investigation comprised different activities (Table 1).

### 2.3. Patient Enrollment in the Study

Each of the PHCUs reviewed and line-listed BCPTB cases that were registered on unit TB registers in the past two years for contact tracing. Those index cases whose contacts were screened and managed previously were excluded, and the remaining line-listed index cases were shared with the HEWs for household visits and subsequent screening of the contacts.

The trained HEWs made household visits to the index BCPTB cases in their respective catchment areas. Once they enumerated and registered all contacts in the household, symptom-based TB screening was conducted for the contacts using a standard national TB screening tool. The HEWs made appointments for those contacts presumed to have active TB and those aged <15 years eligible for TPT to nearby health posts for subsequent re-evaluation and management by the outreach team.

**Figure 1:**
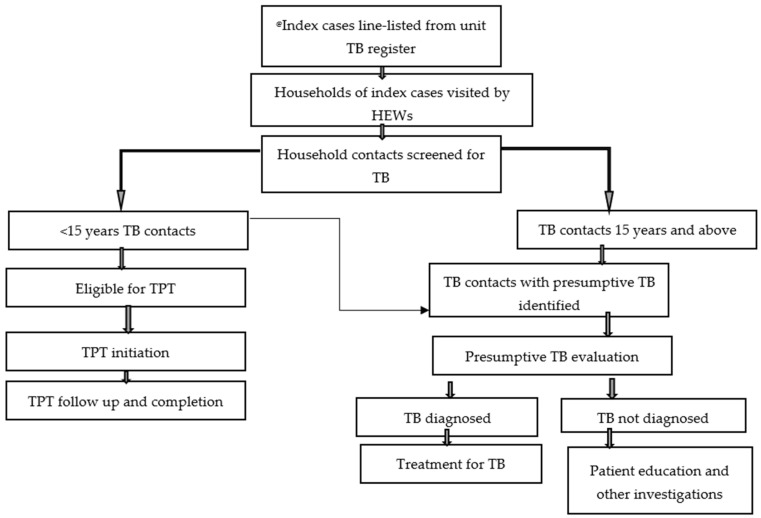
Algorithm for contact screening and TPT cascade in the study. @ Index cases are infectious TB cases or bacteriologically confirmed pulmonary tuberculosis (BCPTB) cases with whom children <15 years had been in contact at least overnight. The TB unit register is the standard register in Ethiopia on which confirmed TB cases are registered while on treatment. Evaluation of contacts resulted in either symptomatic contacts who underwent Xpetr testing or were referred to hospitals for further evaluation, or asymptomatic contacts who were evaluated to start on TB preventive therapy (TPT). Both TPT and anti-TB treatment were followed up for adherence counselling and treatment completion at the community level or health posts.

The outreach team, which comprised district TB officers, facility TB focal persons, and laboratory personnel, provided regular outreach support to the catchment health posts to evaluate the appointed contacts and collect and transport sputum from symptomatic contacts for laboratory testing to GeneXpert sites. Contacts who could not submit sputum were referred to nearby hospitals for clinical evaluation and decision. Those contacts diagnosed with active TB were started on anti-TB treatment per national TB guidelines at health facilities. The TB focal person also assessed asymptomatic under-15-year-old household contacts for TPT eligibility and subsequently counselled family members or caregivers to initiate TPT. Based on the preference of the family members or caregivers, TPT was initiated and followed at either a health post or a health center. Household contacts of BCPTB cases were considered eligible for TPT if they were <15 years, asymptomatic, and had no contraindications for TPT (Figure 1).

The TPT regimen selection was based on the age of the children, availability of the drugs, and preference of caregivers. In many of the health facilities, 3RH was used, which was suitable for all children, while in the health facilities where 3HP was available, it was prescribed for children aged 2 years and above.

### 2.4. Adherence Support and Follow-Up

Once children and adolescents contacts <15 years initiated TPT at health posts, the HEWs provided regular counselling, adherence support, and follow-up monthly. TPT drugs were kept in kit form at the health posts to facilitate drug refill. HEWs assessed TPT completion by asking for the number of missed doses of the prescribed TPT medications. Contacts who successfully took the full course of the prescribed doses were considered to complete their full course of treatment per national guidelines.

### 2.5. Supportive Supervision

Periodic supportive supervision was carried out to oversee and support the health care providers with the implementation of the community-based activities for contact investigation and TPT provision. The performance of community-based contact screening and TPT provision was regularly monitored using contact investigation and the TPT performance tracking tool.

### 2.6. Data Management and Analysis

Data on contact screening and TPT provision were recorded at both the health post (on the contacts investigation log sheet) and health center levels (on the TB contact screening registry). Data from facility registers were checked and cleaned for completeness and consistency. Descriptive statistics were used to analyze and describe the performance data on cascade of contact screening, TB case detection, and TPT uptake and completion among household contacts in the selected PHCUs. Performance data on contact screening and TPT uptake before and after the implementation of enhanced community-based contact screening were compared. TPT uptake and completion were analyzed by age subgroup, type of regimen, and site of TPT service delivery.

The Chi-square test was computed to analyze the categorical data on contact screening and TPT service data during the preintervention and intervention phases using Stata statistical software version 17. Statistical significance was calculated at *p* < 0.05 with a 95% confidence interval (CI).

### 2.7. Ethical Considerations

The study was conducted under the routine programmatic implementation based on national guidelines. Prior permission for use of facility-level routine programmatic data for the purpose of evidence generation and program decision making was obtained from the IRBs of the regional health bureau (reference number of BF/HBOR/1512). The IRB issued a waiver to use TB patients’ data as the data did not have personal identifying information.

## 3. Results

Of the 1069 BCPTB cases enrolled, 1049 households of index cases (98.2%) were visited by the HEWs for contact tracing and screening. Of 4403 contacts of all ages identified, 4367 (99.2%) were screened for TB using a symptom-based checklist during the intervention period. Of these, 2668 (60.6%) were contacts aged 15 years and above, while 1735 (39.4%) were <15 years of age. The contact-to-index case ratio was 4.1:1. The contact screening coverage was 95.6% (394/412) during the preintervention period (Table 2).

**Figure 2:**
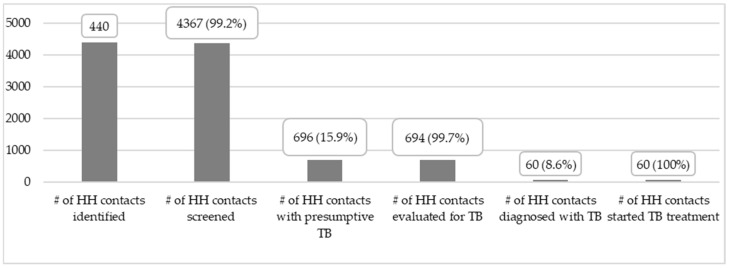
Cascade of care on TB contact screening and TB case detection.

A total of 696 (15.9%) contacts were found to have symptoms of active TB, and 99.7% (694/696) of these were evaluated for TB per the national TB diagnostic algorithm. Sixty (8.6%) household contacts were diagnosed with active TB and initiated on TB treatment during the intervention period (Figure 2). Fifty-six active TB cases (93.3%) were detected among those household contacts aged 15 years and above, while four children <15 years accounted for 6.7% of the contacts diagnosed with active TB.

**Figure 3:**
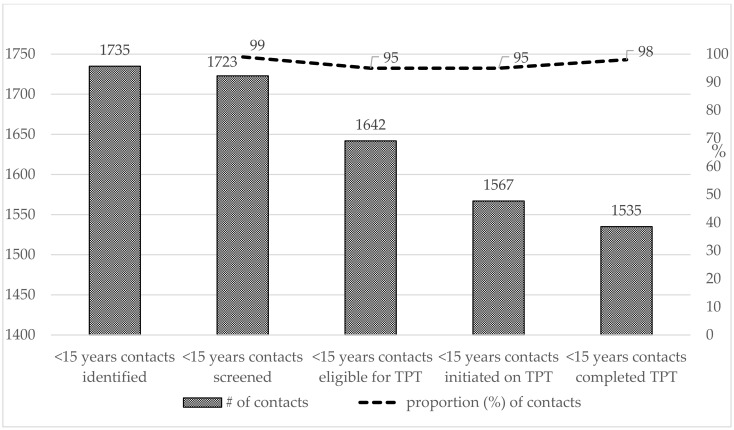
TPT cascade of care among child and adolescent contacts <15 years.

The findings in the TPT cascade among child and adolescent contacts during the intervention are depicted in Figure 3. Of the 1723 <15 years contacts symptomatically screened for active TB, 1642 (95.3%) were negative for TB and eligible for TPT per national TB guidelines. Of these, 1567 (95.4%) children and adolescent contacts <15 years were offered and initiated on TPT, while 98% completed the TPT (Figure 3). Children <5 years accounted for 21% of the total contacts initiated on TPT.

The majority of eligible <15 household contacts (88.8%, 1392/1567) were initiated at the health post level after evaluation and excluding active TB by the outreach team from the catchment health center, while the remaining 175 (11.2%) contacts were started on TPT at the health center level (Table 3). With respect to the TPT regimen prescribed, 1528 (97.5%) contacts were administered 3RH, and 39 (2.5%) received 3HP. The rate of TPT uptake was higher during the intervention period than the preintervention period, and this is statistically significant (81.7% vs. 95.4%; OR, 4.67; 95% CI, 2.54–8.23). The percentages of TPT uptake among contacts <5 years of age and those 5–14 years were 94.0% and 95.7%, respectively. No statistically significant difference was found in TPT uptake between these two groups (94.0% vs. 95.7%; OR, 0.71; 95% CI, 0.41–1.25). During the preintervention period, only two active TB cases were detected. Compared to routine facility-based practice, enhanced community-based interventions significantly increased contact screening coverage (95.6% vs. 99.2%, OR, 5.54; 95% CI, 2.93–10.13) (Table 3 and Table 4).

Findings on TPT completion indicated that, of the 1657 contacts <15 years initiated on TPT during the intervention period, 1537 (98.1%) successfully completed their treatment as prescribed (Table 3). The TPT completion rate significantly increased during the intervention period (92.9% vs. 98.1%; OR, 3.81; 95% CI, 1.28–9.86). There was no difference by age group. The completion rate was similar between 3RH (98.0%) and 3HP (100.0%). There was a difference in completion rate among contacts followed at health posts and health centers (99.0% vs. 88.0%, respectively; OR, 20.95; 95% CI, 8.97–52.71) (Table 4).

## 4. Discussion

In our study, the overall performance in contact tracing and screening in rural PHCUs significantly improved in terms of the number of contacts reached and screened for TB through the implementation of an enhanced community-based contact screening model. The contact screening coverage significantly increased to 99.2%, and 60 contacts were found to have active TB and initiated on standard TB treatment as per national TB guidelines. Similarly, the rates of TPT initiation (95.3%) and completion (98.1%) among eligible <15 years contacts were significantly higher with the community-based intervention than with routine and facility-based service. The TPT completion rate was better for patients followed at health posts than at health centers, indicating a need for decentralized services to the community.

The community-based intervention helped to improve the number of contacts screened by 10-fold as compared to the preintervention period. The contact-to-index case ratio was 4.1:1, which is supported by similar studies showing increased identification of contacts per index case by conducting active contact tracing [20,21,22]. The yield of active TB among contacts was 8.8%, which is higher than in the preintervention period. This finding is supported by several studies that documented increased yield of active TB among household contacts with community-based prospective and retrospective contact screening [23,24,25,26,27,28]. The findings revealed an increased burden of TB among contact BCPTB cases in the rural communities of the study area. Most of the TB cases diagnosed were contacts aged 15 years and above (93.5%), while children <15 years accounted for 6.5% of the cases. This could be explained by the fact that the diagnosis of TB among young children is often difficult due to the challenges associated with sputum collection and testing.

Evidence indicated several limitations associated with facility-based contact screening. Bringing asymptomatic contacts for screening to health facilities and additional cost implications for households pose difficulties to easily accessing contact screening and TPT service [8,9]. Community-based interventions could be a preferable approach to systematically address the challenges and barriers that limit the rural community’s access to and benefits of contact screening and TPT service. Other studies suggest an alternative hybrid approach that provides contact identification and screening in the community with subsequent TB investigations and TPT initiation and follow-up at the health facility [8,9].

Early identification of TB-exposed individuals is an entry point for the TPT cascade of care. A significant number of contacts are missed before visiting clinics for screening, evaluation, and subsequent TPT initiation [29,30]. In our study, the overall rate of TPT uptake among eligible <15 years contacts was 95.3%, which was significantly higher than the preintervention period. It was found that 94.0% of child contacts <5 years and 95.7% of those 5–14 years were initiated on TPT. In our study, there was no statistically significant difference in the uptake of TPT among children <5 years of age and those between 5 and 14 years (94.0% vs. 95.7%). Our finding is supported by other studies that reported higher rates of TPT uptake among household contacts at the community level in rural health facilities in Africa, ranging from 91.0 to 98.0% [15,31,32,33]. TPT uptake tends to increase by around 5-fold with an enhanced community-based approach compared to a facility-based approach, which could be explained by TPT service delivery happening at the community level. Several studies support our finding of improved TPT uptake and completion with a community-based contact investigation approach. For example, a multi-country study conducted in Benin, Burkina Faso, Cameroon, and the Central African Republic reported a TPT initiation rate of 91% and a completion rate of 94% [32,33]. A novel community-based TB contact management program in Eswatini found that 98% of eligible asymptomatic household contacts were initiated on TPT, and 93% completed TPT at the community level [15,16].

Adherence support and completion of TPT are critical for better outcomes among contacts [7]. In this study, 1537 (98.2%) household contacts <15 years successfully completed their full course of TPT with the enhanced community-based approach. Of these, 90% of the <15 years contacts were followed and received adherence support at the health post level by trained HEWs. Our findings show that household contacts who received treatment adherence support at the health post level were more likely to complete their treatment than those who had facility-based follow-up. The TPT completion rate among contacts aged <15 years was very high, and no significant difference was observed between younger and older children. The finding of a TPT completion rate above 90% at the community level was comparable with studies conducted in Ethiopia, Eswatini, and other African countries [15,16,23,25,33]. This could be explained by the provision of ongoing family education and counselling on the importance of TPT by trained HEWs and improved access to and follow-up of TPT services at the community level. Moreover, more than 98.0% of the household contacts were initiated on 3RH, a child-friendly short course regimen that has been found to promote better treatment acceptance and adherence, resulting in a higher completion rate [8,9,34]. Above all, there have been no significant concerns from the health care providers regarding the risk of resistance to the drugs, which could be due to the fact that the job aids and training materials provided evidence of a very low risk of emerging resistance due to TPT.

Access to contact screening and TPT service delivery can potentially be facilitated by active contact tracing and decentralizing patient-centered contact management services to the community level. This would help to avoid several barriers and challenges that prevent the community from accessing these services [9,20]. Our findings also show that a decentralized, community-based prospective and retrospective contact screening strategy through engagement of HEWs facilitated reaching contacts who could have missed the opportunity to initiate TPT through the routine standard of care in health facilities. This finding highlights the need to adopt context-specific strategies to decentralize access to contact screening and TPT service at the community level, which might be strategically important to have a meaningful impact on TB prevention in high-burden areas. With the current opportunities in policy guidelines supporting the expansion of TPT access to children and adolescent contacts, and the wider availability of shorter TPT regimens in Ethiopia, resource allocation to support the community system and capacity building efforts for task shifting are vital to decentralize contact investigation and TPT services in the community beyond the health facility.

Although this study was conducted under a routine program context and was easy to integrate and carry out using existing structures in the community, it has some limitations. First, the study was implemented in high TB volume rural PHCUs and did not include facilities with a medium patient load or facilities in urban areas. The modality of community-based initiatives to address household contact screening and TPT uptake for urban communities needs to be further studied. Second, contacts who had initial negative test results following evaluation for TB were not followed up and reassessed for clinical TB cases or TPT eligibility and initiation. Thus, only BCPTB cases were reported, which might undermine real TB cases among contacts. Also, the rate of TPT uptake was analyzed only from contacts who had a negative TB result at initial screening. Third, information on completion of a course of TPT was obtained through self-reporting by family members or caregivers during monthly visits for drug refills at health posts and health centers. We did not implement other means of monitoring the adherence of contacts initiated on TPT in the study. Thus, the study could potentially be influenced by the disadvantages associated with self-reporting. This requires further evaluation for a better understanding of adherence rates of household contacts taking TPT and determinants in the rural community. Fourth, TB screening among household contacts was carried out entirely by evaluating symptoms, and we did not use more sensitive screening tools like chest X-rays. Hence, potential presumptive TB cases could be missed and initiated on TPT because they were asymptomatic. However, there were no case reports of active TB and discontinuation of treatment for clients on TPT. In the end, only the gross age category (<15 years and >15 years) was used, and we could not collect contacts and TPT data based on detailed age categories. The analysis focused on before and after interventions, and only two variables were used where multivariable analysis was not conducted to deal with confounding factors.

## 5. Conclusions

In this study, the systematic implementation and monitoring of community-based contact screening with engagement of trained HEWs remarkably increased the TPT uptake and completion of eligible children and adolescent household contacts in high-TB-burden rural PHCUs. Decentralized and active contact tracing through an enhanced community-based model of interventions should be supported and scaled up to other high-burden districts and health facilities to increase access to TPT for eligible household contacts in resource-constrained settings.

## Figures and Tables

**Table 1:** Key interventions, monitoring, and support.

Key Interventions ^#^	Preintervention (Routine Practice)
1.0 Capacity building	
Customized training materials on contact investigation and TPT provision were developed. District-level one-day orientation training was provided to PHCU teams (district TB officers, PHCU directors, facility TB focal persons, and HEWs). Standard operating procedures were provided for the health facilities. Simplified job aids were printed and distributed to the health facilities.	Contact investigation and TPT training were integrated into basic TB and TB/HIV training. Basic TB and TB/HIV training were provided predominantly to district and facility TB focal persons. Standard operating procedures were not readily available. Job aids on contact investigation and TPT were not routinely available at health facilities.
2.0 Contact screening and evaluation practice	
Community-based active contact screening conducted at household level by trained HEWs. Trained HEWs carried out household visits for enumerating and screening contacts. Outreach supports to re-evaluate TB contacts with presumptive TB and those <15 years at health posts. Collection and transportation of sputum for GeneXpert testing by outreach team whenever possible.	Passive contact tracing and screening with referral of eligible contacts was the routine practice at health centers. Index TB cases were counselled by TB focal persons to bring TB contacts to the health facility. No outreach support activity to the community. No sputum collection or transportation from the community; TB contacts requiring evaluation for TB and TPT initiation were directly referred to the health centers.
3.0 TPT initiation and follow-up	
Eligible <15 years TB contacts were initiated on TPT after counselling at health posts and health centers. TPT follow-up and adherence support were performed at the health post level by trained HEWs.	TPT was initiated to eligible <15 years TB contacts solely at health centers. TPT follow-up was carried out by trained TB focal persons at health centers.
4.0 Program monitoring and support	
Regular onsite mentoring and supportive supervision provided to health centers and health posts. Performance tracking tool for contact investigation and TPT introduced. Data recorded on TB contact screening registration at health centers. Data recording tool provided to health posts. Frequent monitoring of contact investigation and TPT data using tracking tool. Feedback provided to HEWs based on observed gaps to maintain the quality of the service. A communication platform phone and telegram were arranged for regular communication and consultation to address unclear issues	Infrequent integrated supportive supervision provided to health centers. No performance tracking tool. Data were recorded on TB contact screening registration at health centers. No data recording tool at health posts. Routine quarterly data reporting and monitoring performed using DHIS2.

# The table compares interventions at the selected districts for outreach contact investigation and TPT to the routine national implementation. There are four key interventions: (1) training of HEWs and health care providers; (2) outreach or community-based contact investigation; (3) TPT initiation; and (4) monitoring of these activities through regular mentorship and supportive supervision. Note: DHIS2 stands for District Health information system; TPT refers to TB Preventive Therapy, and HEWs to health extension workers (community health workers).

**Table 2:** Baseline characteristics of household contacts screened and evaluated for TB.

Variables	N (%)
Age (*n* = 4403)	
<15 years	1735 (39.4)
≥15 years	2668 (60.6)
Period of enrolment	
Intervention	4403
Preintervention	412
Cascade of contact screening	
Index bacteriologically confirmed PTB cases	1069
Households visited	1050 (98.2)
Contacts screened for TB (*n* = 4374)	
<15 years	1723 (39.4)
≥15 years	2651 (60.6)
Contacts with positive TB screen result (*n* = 696)	
<15 years	81 (11.6)
≥15 years	615 (88.4)
Contacts evaluated for TB (*n* = 687)	
<15 years	79 (11.4)
≥15 years	608 (88.6)
Contacts diagnosed with active TB (*n* = 60)	
<15 years	4 (6.7)
≥15 years	56 (93.3)
Contacts put on TB treatment (*n* = 60)	
<15 years	4 (6.7)
≥15 years	56 (93.3)

**Table 3:** Characteristics of participants by TPT initiation and completion.

Variables	N (%)
TPT initiation	
Age	
<5 years	328 (20.9)
5–14 years	1239 (79.1)
Total	1567 (100.0)
Period of enrolment	
Intervention	1567 (95.4)
Preintervention	85 (92.9)
Type of TPT regimen (*n* = 1567)	
3RH	1528 (97.5)
3HP	39 (2.5)
Site of TPT initiation (*n* = 1567)	
Health center	175 (11.2)
Health post	1392 (88.8)
Total	
TPT completion	
Age (*n* = 1537)	
<5 years	320 (20.8)
5–14 years	1217 (79.2)
Period of enrolment	
Intervention	1537 (98.1)
Preintervention	79 (95.4)
Type of TPT regimen (*n* = 1537)	
3RH	1498 (98.0)
3HP	39 (100.0)
Site of adherence support (*n* = 1537)	
Health post	1383 (99.4)
Health center	154 (88.0)

**Table 4:** Characteristics of factors affecting contact screening, TPT initiation, and completion.

Variables	Yes (*n*, %)	No (*n*, %)	OR	95% CI	*p*-Value
Contacts screened for TB					
Preintervention	394 (95.6)	18 (4.4)	5.54	2.93–10.13	<0.001
Intervention	4367 (99.1)	36 (0.9)			
TB diagnosed among contacts					
Preintervention	2 (8.3)	22 (91.7)	1.06	0.25–9.54	0.93
Intervention	60 (8.8)	621 (91.2)			
TPT initiation					
<5 years	328 (94.0)	21 (6.0)	0.71	0.41–1.25	0.18
5–14 years	1239 (95.7)	54 (4.3)			
Preintervention	85 (92.9)	19 (7.1)	4.67	2.54–8.23	<0.001
Intervention	1567 (98.1)	75 (1.9)			
TPT completion					
<5 years	320 (97.6)	8 (2.4)	0.72	0.31–1.90	0.44
5–14 years	1217 (98.2)	22 (1.8)			
Intervention	1537 (98.1)	30 (1.9)	3.89	1.28–9.86	0.002
Preintervention	79 (95.4)	6 (4.6)			
Adherence at health post	1383 (99.4)	9 (0.6)	20.95	8.97–52.71	<0.001
Adherence Health Center	154 (88.0)	21 (12.0)			

## Data Availability

All data used here are available in the manuscript.

## Abbreviations

The following abbreviations are used in this manuscript:

DOTs	Directly observed treatment
DR-TB	Drug-resistant TB
HEWs	Health extension workers
3HP	Rifapentine and isoniazid
PHCUs	Primary health care units
SNNPR	Southern Nations, Nationalities, and Peoples’ Region
PTB	Pulmonary tuberculosis
TB	Tuberculosis
TPT	Tuberculosis preventive treatment
